# CT-based radiomics combined with signs: a valuable tool to help radiologist discriminate COVID-19 and influenza pneumonia

**DOI:** 10.1186/s12880-021-00564-w

**Published:** 2021-02-17

**Authors:** Yilong Huang, Zhenguang Zhang, Siyun Liu, Xiang Li, Yunhui Yang, Jiyao Ma, Zhipeng Li, Jialong Zhou, Yuanming Jiang, Bo He

**Affiliations:** 1 Medical Imaging Department, First Affiliated Hospital of Kunming Medical University, Kunming, 650000 China; 2 Precision Health Institution, PDx, GE Healthcare (China), Beijing, 100176 China; 3 Department of Radiology, The 3rd Peoples’ Hospital of Kunming, Kunming, 650000 China; 4 Department of Medical Imaging, People’s Hospital of Xishuangbanna Dai Autonomous Prefecture, Xishuangbanna, 666100 China; 5 Medical Imaging Department, Yunnan Provincial Infectious Disease Hospital, Kunming, 650000 China; 6 MRI Department, The First People’s Hospital of Yunnan Province, Kunming, 650000 China

**Keywords:** Coronavirus disease 2019, Viral pneumonia, Radiomics, X-ray computed tomography

## Abstract

**Background:**

In this COVID-19 pandemic, the differential diagnosis of viral pneumonia is still challenging. We aimed to assess the classification performance of computed tomography (CT)-based CT signs and radiomics features for discriminating COVID-19 and influenza pneumonia.

**Methods:**

A total of 154 patients with confirmed viral pneumonia (COVID-19: 89 cases, influenza pneumonia: 65 cases) were collected retrospectively in this study. Pneumonia signs and radiomics features were extracted from the initial unenhanced chest CT images to build independent and combined models. The predictive performance of the radiomics model, CT sign model, the combined model was constructed based on the whole dataset and internally invalidated by using 1000-times bootstrap. Diagnostic performance of the models was assessed via receiver operating characteristic (ROC) analysis.

**Results:**

The combined models consisted of 4 significant CT signs and 7 selected features and demonstrated better discrimination performance between COVID-19 and influenza pneumonia than the single radiomics model. For the radiomics model, the area under the ROC curve (AUC) was 0.888 (sensitivity, 86.5%; specificity, 78.4%; accuracy, 83.1%), and the AUC was 0.906 (sensitivity, 86.5%; specificity, 81.5%; accuracy, 84.4%) in the CT signs model. After combining CT signs and radiomics features, AUC of the combined model was 0.959 (sensitivity, 89.9%; specificity, 90.7%; accuracy, 90.3%).

**Conclusions:**

CT-based radiomics combined with signs might be a potential method for distinguishing COVID-19 and influenza pneumonia with satisfactory performance.

**Supplementary Information:**

The online version contains supplementary material available at 10.1186/s12880-021-00564-w.

## Background

In December 2019, a highly infectious disease caused by severe acute respiratory syndrome coronavirus 2 (SARS-CoV-2) infection broke out in Wuhan, China, known as coronavirus disease 2019 (COVID-19) [1, 2]. COVID-19 is still spreading around the world at an alarming rate. Early studies have shown that almost all COVID-19 patients have pneumonia [3, 4]. However, pneumonia caused by influenza pneumonia is also very common at the same period of year, and clinical symptoms are very similar [5–7]. And early diagnosis is of great significance to the prognosis of COVID-19 patients, especially the occurrence of adverse events. Liu et al. found that quantifying lung lesions early (0–4 days) can predict the severity of the disease [8]. Therefore, in this COVID-19 pandemic, the differential diagnosis between COVID-19 and influenza pneumonia is difficult but highly important in the early stages of the disease. Real-time reverse transcription-polymerase chain reaction (RT-PCR) is the gold standard for the diagnosis of viral pneumonia. However, recent reports have shown that RT-PCR detection of COVID-19 has low sensitivity [9], and the high false-negative rate limits the rapid identification of viral pneumonia by RT-PCR.

Currently, computed tomography (CT) can play an important role in the diagnosis and treatment of viral pneumonia [10, 11]. Studies have shown that the CT signs of COVID-19 and influenza pneumonia are different [12, 13]. However, little is known about the prediction performance of CT signs in distinguishing COVID-19 from influenza pneumonia in previous studies. And radiologists subjectively evaluate viral pneumonia through imaging signs, and the accuracy of diagnosis depends on the doctor's diagnosis experience. Therefore, it is also necessary to further develop a rapid quantitative auxiliary diagnostic method to identify COVID-19 and influenza pneumonia.

With the rapid development of computer technology, medical image processing technology is widely used in the diagnosis and treatment of COVID-19. Xu et al. used multiple CNN models to classify CT images of normal, COVID-19 and influenza A pneumonia, with accuracy ranging from 71.8% to 85.0% [14]. Radiomics is a new quantitative analysis technology based on medical imaging, which could extract thousands imaging features including first-order statistical, shape, second- or higher order texture features. Compared with deep learning models, radiomics which is based on the mathematical description of image texture might be more interpretable, and can further help understanding their physiopathology by correlating with laboratory or pathological information. Previous studies have shown that radiomics has outstanding performance in tumor diagnosis, treatment effect evaluation, and prognosis prediction [15–17]. Recently, there had already been some constructed radiomics model based on deep-learning to predict the prognosis of COVID-19 patient [18]. Chen et al. and Wu et al. found that radiomics model based on CT images is a feasible and promising method for monitoring poor prognostic outcome (acute respiratory distress syndrome, death, need for mechanical ventilation, or intensive care unit admission) in patients with COVID-19 [19, 20]. Besides, radiomics has been also used to identify focal organizing pneumonia and peripheral lung adenocarcinoma [21]. However, whether independent model or combined model of CT signs and radiomics can help radiologist distinguish identify COVID-19 and influenza pneumonia and improve the diagnostic performance still remains unclear.

Therefore, in present study, we aim to select significant chest CT signs and radiomics features that can effectively identify COVID-19 and influenza pneumonia, and determine whether a CT-based radiomics signature combined with CT signs could be used as a tool in the differentiation of COVID-19 and influenza pneumonia.

## Methods

### Patients

**Fig. 1:**
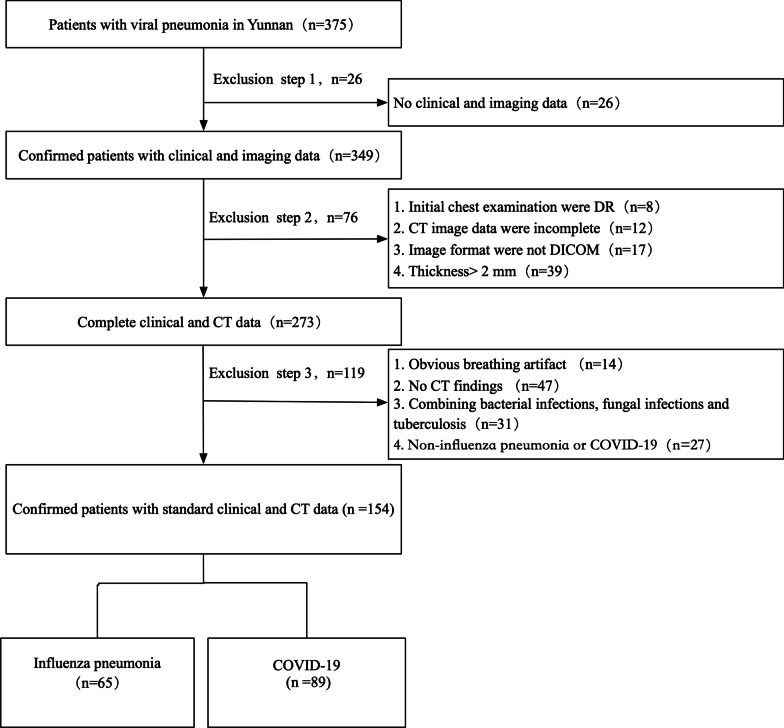
Flow diagram of the study design and patient enrollment in the analysis

**Fig. 2:**
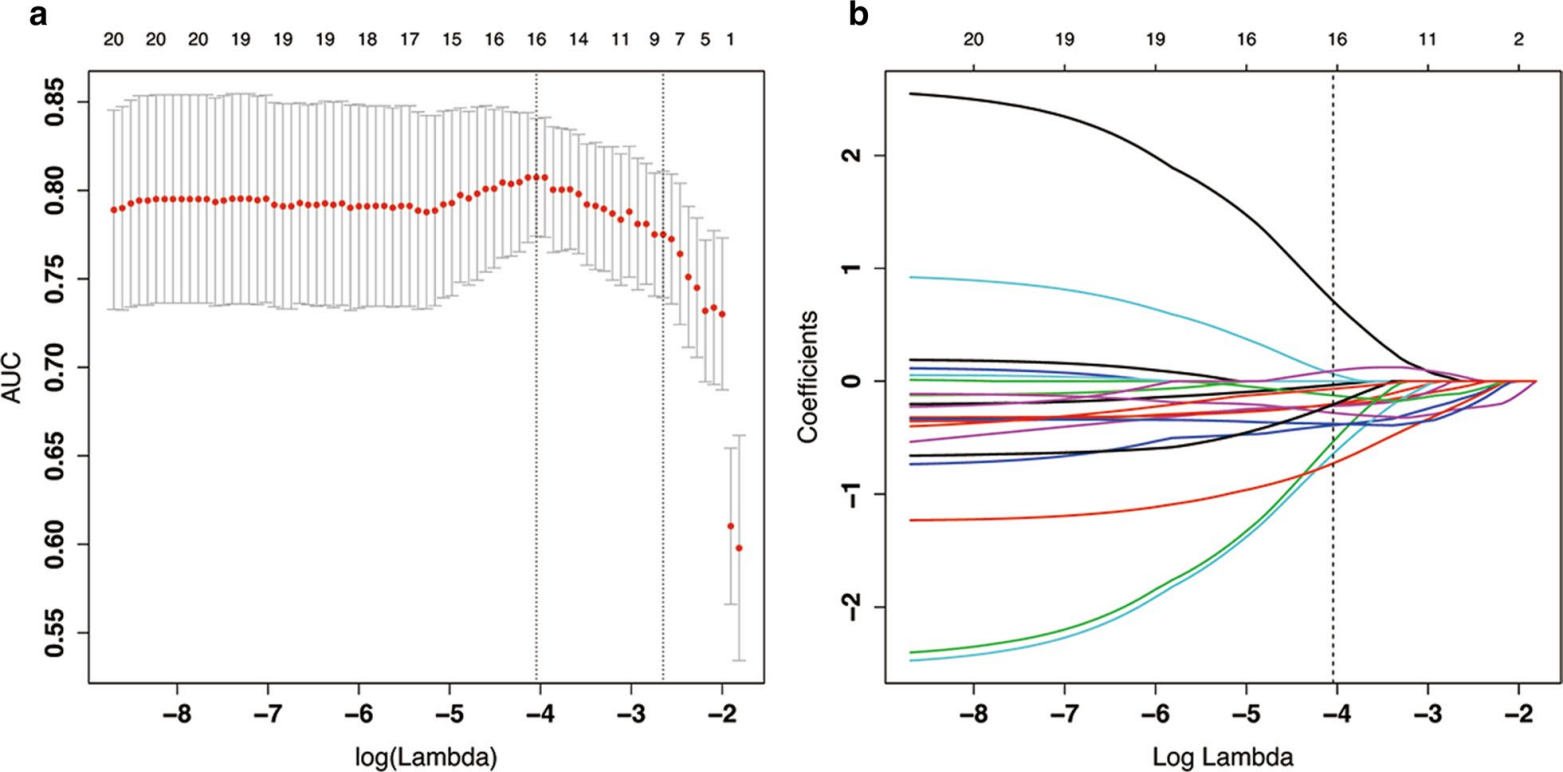
The radiomic feature selection using the least absolute shrinkage and selection operator (LASSO) binary logistic regression model. **a** Parameter (Lambda) is tuned in the LASSO model using fivefold cross-validation via maximum area under the ROC curve criteria. AUC on each fold was drawn versus log (Lambda). Vertical line was drawn at the determined optical log (Lambda) with one standard error among fivefold cross-validation, where optimal Lambda resulted in nonzero coefficients. **b** A coefficient profile plotted against the log (Lambda)

This retrospective study was approved by our institutional review board and patient consent was waived. This study retrospectively collected patients with viral pneumonia diagnosed in 15 hospitals in this province from March 1, 2015 to March 15, 2020. RT-PCR assays were performed to identify influenza A virus, influenza B virus, respiratory syncytial virus, parainfluenza virus, adenovirus, SARS coronavirus, SARS-CoV-2, Epstein-Barr virus, measles virus, and other viruses from nasopharyngeal swabs or bronchoalveolar lavage fluid. The study only included pneumonia patients infected with single virus, and patients with multiple respiratory viruses or bacterial or fungal infections were excluded. A total of 375 viral pneumonia patients were diagnosed in this study. The further selection process for viral pneumonia patients is shown in Figs. 1, 2. All patients were admitted within 7 days after the onset of acute symptoms and completed the chest CT examination within 48 h after admission. According to the virus type found in the lungs, the patients were divided into two groups: COVID-19 and influenza pneumonia. The number of cases included in each hospital is summarized in Additional file 4: Table 1.

**Table 1:** The predicting performance for CT signs model, radiomics model and combined model

Model	CT signs	Radiomics	CT signs + radiomics
Threshold	0.431	0.107	0.458
AUC (95% CI)	0.906 (0.86–0.953)	0.888 (0.8335–0.9417)	0.959 (0.930–0.987)
Sensitivity (95% CI)	0.865 (0.7774 to 0.9227)	0.865 (0.7774 to 0.9227)	0.899 (0.8168 to 0.9479)
Specificity (95% CI)	0.815 (0.7029 to 0.8927)	0.784 (0.6691 to 0.8683)	0.907 (0.8096 to 0.9603)
Positive prediction (95% CI)	0.865 (0.7774 to 0.9227)	0.846 (0.7569 to 0.9074)	0.930 (0.8532 to 0.9705)
Negative prediction (95% CI)	0.815 (0.7029 to 0.8927)	0.809 (0.6944 to 0.8891)	0.868 (0.7650 to 0.9310)
Accuracy (95% CI)	0.844 (0.7780 to 0.8936)	0.831 (0.7636 to 0.8826)	0.903 (0.8445 to 0.9411)

AUC, area under curve

### CT examination

HRCT examination: CT scanners with 16 or more detector rows (Siemens, Germany; Philips, the Netherlands; and GE, USA) were used. The patient was scanned in the supine position while holding his or her breath after inspiration. The scanning range was from the thoracic inlet to the costophrenic angles. Scanning parameters: detector collimation width 64 × 0.6 mm or 128 × 0.6 mm, tube voltage 120 kV, adaptive tube current, high-resolution algorithm reconstruction, reconstruction layer thickness 1 or 1.5 mm, and layer spacing 1.5 mm.

### Chest CT signs analysis

Three Chinese radiologists were blinded to the RT-PCR results, all patient information, and type of viral pneumonia. First, two experienced thoracic radiologists independently read the CT images. When their opinions were inconsistent, they discussed them and reached a consensus, which was reviewed and confirmed by the third senior radiologist in the cardiothoracic group. The signs of the first CT examination after admission were analyzed. The CT imaging evaluation included lesion distribution (central, peripheral and mix), location (left upper lobe, left lower lobe, right upper lobe, right middle lobe and right lower lobe) and main signs [GGO (ground-glass opacities), partial consolidation, consolidation, septal thickening, intralobular interstitial thickening, crazy-paving pattern, tree-in-bud, bronchial wall thickening, bronchiectasis, air bronchogram, halo sign, reversed halo sign, mediastinal lymphadenectasis, and pleural effusion] [10–12, 22]. The window width and level were set to 1600/-600 HU.

### CT image processing and volume of interest (VOI) segmentation

The Lung Kit software (GE Healthcare, Version LK2.2) was used for pneumonia lesion segmentation. All the CT images were firstly resampled into isotropic 1 mm × 1 mm × 1 mm voxel size using trilinear interpolation, to reduce the impact of different scanner and scanning parameters. Then, the images were processed by low-pass Gaussian filter to increase the reproducibility of the radiomics features to be extracted [23]. During segmentation process, the five anatomic lung lobes were firstly automatically segmented by using Dense V-networks to further help positioning the pneumonia lesions. Next, the lesion segmentation was fulfilled by computer–human collaboration. The pneumonia lesions which distributed in the whole lung was firstly segmented automatically and considered as one integrated 3-dimensional volume of interest (VOI) by the LK software [24]. And then one experienced thoracic radiologist (ZG Zhang, 5 years experience in chest imaging) checked the segmentation and made any correction manually if necessary. The resulted VOIs were double checked by another senior radiologist (B He, 15 years experience in chest imaging). The final VOIs were determined until the two radiologists reached consensus. Such segmented lesion VOI was used for radiomics feature extraction in the next step.

### Radiomics feature extraction

A total of 1316 radiomics features were extracted based on the processed CT images and segmented VOIs by using open source of Python package Pyradiomics [25] at gray-level discretization bin width = 25 HU [26]. The extracted radiomics features were categorized into five groups: (1) First-order features including 18 intensity statistics and 14 shape features; (2) 75 multi-dimensional texture features including 24 Gy Level Co‐occurrence Matrix (GLCM), 16 Gy Level Size Zone Matrix (GLSZM), 16 Gy Level Run Length Matrix (GLRLM), 14 Gy Level Dependence Matrix (GLDM) and 5 Neighboring Gray Tone Difference Matrix (NGTDM) Features; (3)1209 Transformed first-order and textural features including: 744 wavelet features in frequency channels LHL, LLH, HHH, HLH, HLL,HHL, LHH and LLL; 186 Laplacian of Gaussian (LoG) filtered features with sigma of 2.0 and 3.0; 279 local binary pattern (LBP) filtered texture features.

### Feature selection and model construction

The whole dataset (n = 154) was used for feature selection and model construction by considering the limited sample size. The radiomics feature data was firstly preprocessed by replacing missing values with median values, and z-score normalization was followed. Next, the features with identical value or near-zero variance were excluded and redundant collinear features were reduced by correlation analysis at a cut-value of 0.7. In addition, Mann–Whitney *U* test was applied to select the features with significant difference (*P* < 0.05) between the COVID-19 and influenza groups. To further reduce the redundancy and complexity of the model, minimum redundancy maximum relevance (mRMR) was performed for feature selection [27, 28] and 20 important features were retained. By considering the sample composition ratio (negative:65, positive:89), the least absolute shrinkage and selection operator (LASSO) logistic regression method with fivefold cross validation was applied for further feature selection and regularization to improve the model accuracy and avoid overfitting. The maximum area under ROC curve for model fitting among the 5 folds was utilized to determine the optimized lambda values. Finally, the remaining features with non-zero coefficients were involved into multi-variate backward stepwise logistic regression with minimum AIC (Akaike Information Criterion) method for model construction. The radiomics signature “Radscore” was produced based on the regression coefficients. Besides, in order to test the reliability of the selected features and the logistic regression model, the 1000-times bootstrap [29] was conducted to obtain the feature appearing frequency and the model’s overall performance.

The independent predictors among CT signs were selected by using Chi-square test (or Fisher exact test), univariate and backward stepwise multivariate logistic regression methods [30–33]. Firstly, the CT signs with P-value less than 0.05 in Chi-square (or Fisher exact test) were retained to be further conducted with univariate and multivariate logistic regression. After sequentially selected by univariate (*P* < 0.05) and multivariate logistic regression, the independent predictors in differentiating COVID-19 and influenza were selected. These finally selected CT signs were used to construct “CT sign” model using logistic regression method.

In addition, the retained CT signs after univariate logistic regression were mixed with radiomics Radscore to be further selected by backward stepwise multivariate logistic regression methods with minimum AIC. The “combined model” was constructed based on the retained features in the last step by using logistic regression method. The nomogram of such combined model was also established.

The classification performances of the radiomics, CT sign and combine models were evaluated by receiver operating characteristic (ROC) curve. The area under the curve (AUC), accuracy, sensitivity and specificity were derived. In addition, the calibration curves and decision curve analysis (DCA) curves were calculated to assess the models’ calibration and their clinical benefits. In addition, the reliability of the selected features and logistic regression model was tested by using 1000-times bootstrap among the whole dataset (detailed steps are described in Additional file 5: Material 2).

### Statistical analysis

The continuous variables or ordinal variables were compared by t-test or Mann–Whitney U test. The distribution of different CT signs was compared by Chi-squared test or Fisher exact test when small sample sizes existed. For ROC analysis, the cut-off value in the training set at the maximum of Youden index of each model was calculated and the confusion matrix and sensitivity, specificity, accuracy in the training and test cohorts were derived at such cut-off value. The Delong test was used for comparison of ROC curves between different models. The reported statistical significance levels were all two-sided with the statistical significance set as *P* < 0.05. The statistical analyses were performed with SPSS Software (Version 25, IBM, Chicago, IL) and R software (Version: 3.6.1, https: www.r-project.org). The following R packages were mainly involved including: “glmnet” for logistic regression including LASSO regression; “pROC” for ROC analysis; “rmda” for DCA analysis.

## Results

### Chest CT signs of viral pneumonia and their predictive performance

**Fig. 3:**
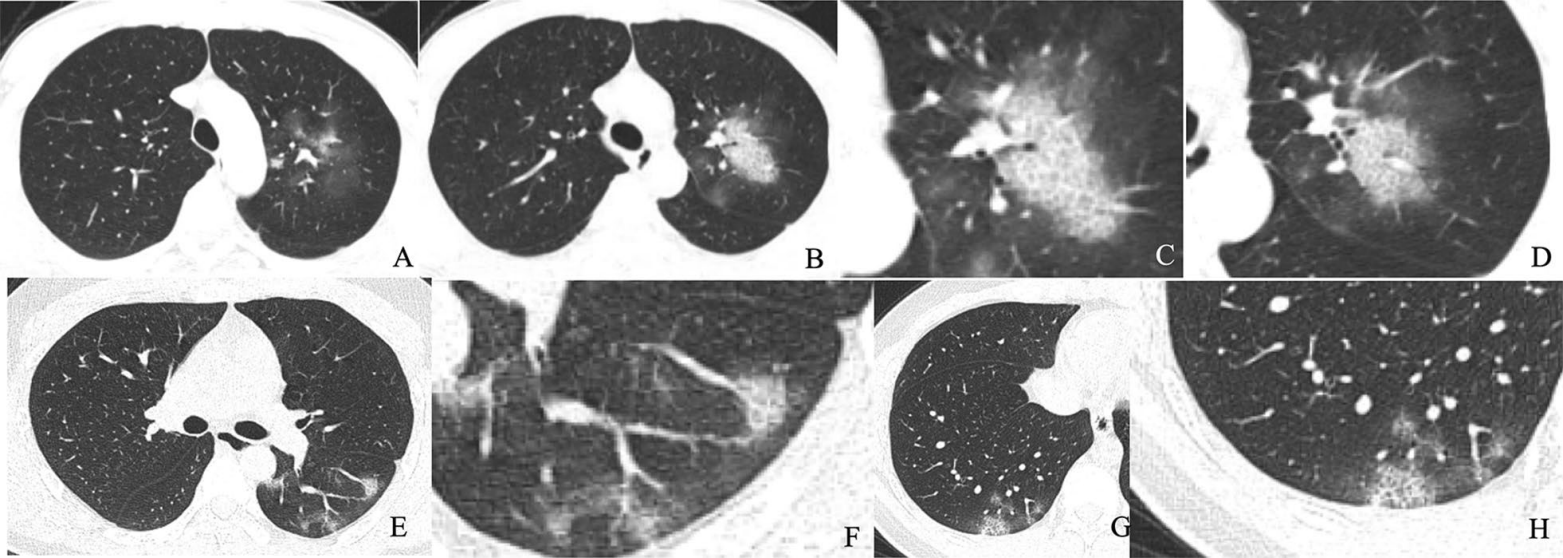
Chest CT findings of COVID-19 pneumonia. **a**–**d** Male, 28 years old, 3 days after onset, axial view by CT scan. Multiple pure GGO in the upper left lobe (**a**, **b**), intralobular interstitial thickening and halo sign (**c**), vascular thickening and halo sign (**d**). E–H: Male, 25 years old, 2 days after onset, axial view by CT scan. Multiple pure GGO in the lower of both lobe (**e**, **g**), vascular thickening and halo sign (**f**); intralobular interstitial thickening and halo sign (**h**)

**Fig. 4:**
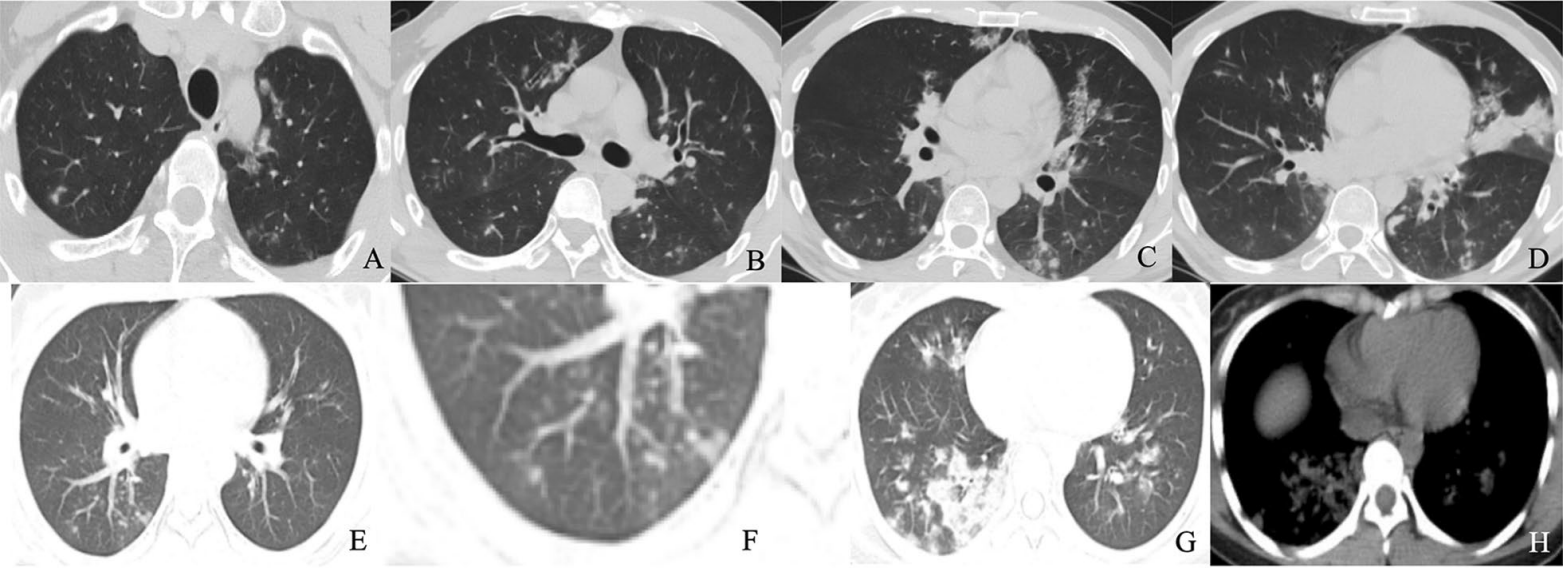
Chest CT findings of influenza pneumonia. Male, 35 years old, 4 days after onset. Multifocal GGO, partial consolidation and consolidation in both lungs (**a**–**d**). Feale, 39 years old, 6 days after onset. Multifocal GGO, partial consolidation and consolidation in the right lower lungs (**e**, **g**). tree-in-bud (**f**), and consolidation (**h**)

This study included 154 patients with viral pneumonia (male: 80; female: 74. Mean age: 44.89 ± 13.85), including 89 cases of COVID-19 pneumonia and 65 cases of influenza pneumonia. The 16 CT signs of COVID-19 and influenza pneumonia were gradually screened by using Chi-square test or Fisher exact test (Additional file 5: Table 2), univariate and multivariate logistic regression analysis (Additional file 6: Table 3). Figures 3, 4 show representative cases of COVID-19 and influenza pneumonia. Four independent predictors were selected, including lesion distribution, GGO, intralobular interstitial thickening, and halo sign. The CT signs model was constructed by using these independent predictors and the performance of the model was summarized in Table 1.

**Table 2:** 1000-times Bootstrap estimate of the area under the ROC curve and the model optimism estimation for the radiomics and CT sign models

Model	Apparent AUC^a^	AUC Bootstrap-Train^b^ (mean, 95% CI)	AUC Bootstrap-Test^c^ (mean, 95% CI)	Average optimism^d^	Optimism-corrected AUC^e^
Radiomics	0.888	0.899 (0.897–0.901)	0.838 (0.835–0.841)	0.061	0.827
CT sign	0.906	0.908 (0.907–0.910)	0.888 (0.886–0.890)	0.020	0.886

^a^The AUC of predicting model developed in original whole dataset

^b^The averaged model performance in the resampled training set after 1000-times bootstrap

^c^The averaged model performance in the r “out-of-bag” test set after 1000-times bootstrap

^d^The model’s averaged optimism as the difference between the bootstrap training set AUC and the test AUC

^e^The corrected AUC by subtracting the average optimism from the apparent AUC

### Extraction and selection of radiomics features and building of the radiomics prediction model

After the near-zero variance, colinear feature reduction (cut-value 0.7) and univariant Mann–Whitney U test (*P* < 0.05), there were 57 features retained. By using mRMR method, the selected 20 important features were conducted with LASSO logistic regression with fivefold cross validation. As shown in Fig. 2, the optimized lambda value (0.017) was chosen at which the maximum AUC 0.807(95% CI 0.774–0.841) for model fitting among the 5 folds was obtained. And 16 features were retained at such lambda value. Furtherly, the backward stepwise logistic regression was utilized to select the final 7 features to construct the logistic regression model.

**Fig. 5:**
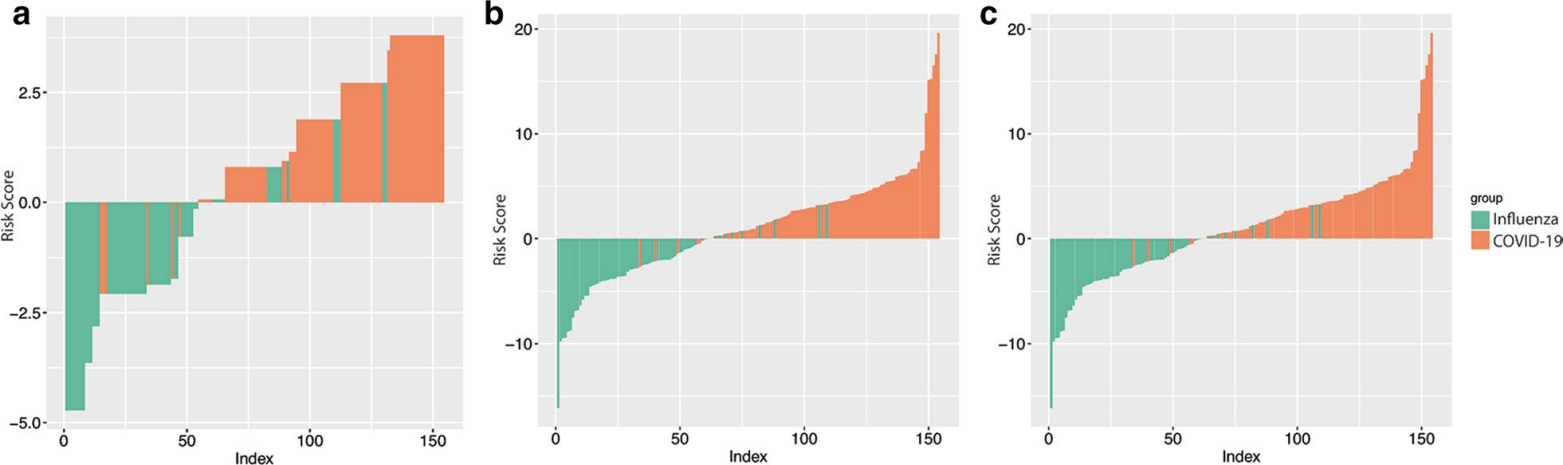
The bar chart of three models for patients with COVID-19 and influenza pneumonia. **a** CT signs model; **b** radiomics model; **c** combined model

Features dimension reduction was performed on 1316 radiomics features and 11 reliable imaging features were finally selected to identify COVID-19 from influenza pneumonia. The statistical difference of each radiomics feature between COVID-19 and influenza pneumonia and their decriptions are summarized in Additional file 7: Table 4. The risk score formula of the radiomics model, CT sign model and the combined model were calculated by using logistic regression coefficients and are listed in the Additional file 2: Material 1. Distributions of the risk score of each model and types of viral pneumonia in the whole database are shown in Fig. 5.

### Predictive performance of the radiomics model, CT signs model and combined model

**Fig. 6:**
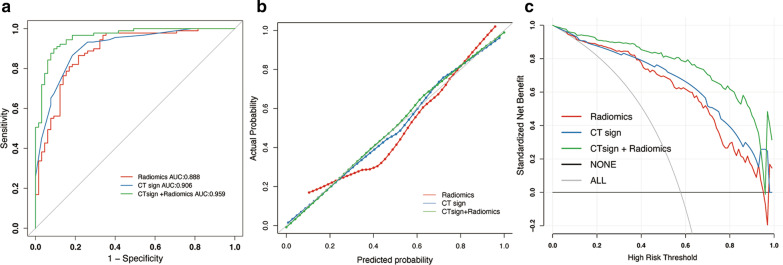
Predictive performance of the CT signs model, radiomics model and the combined model. **a** Receiver operating characteristic (ROC) curves. **b** Calibration curves. **c** Decision curve analysis

**Fig. 7:**
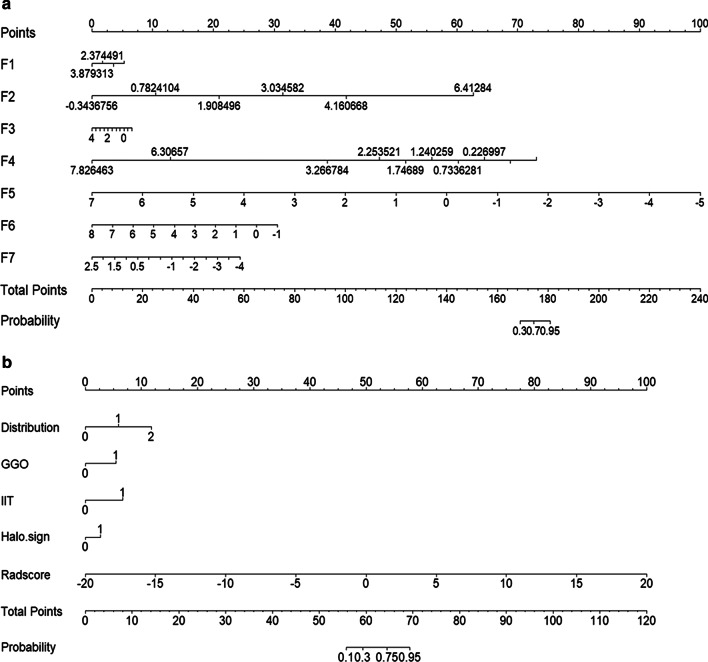
The nomogram representing radiomics model and combined model. **a** Radiomics model; **b** combined model. F1: "lbp.3D.k_ngtdm_Contrast"; F2: "lbp.3D.k_ngtdm_Strength"; F3:"lbp.3D.m2_glszm_SmallAreaEmphasis"; F4: "wavelet.LLH_ngtdm_Contrast"; F5:"wavelet.HLL_firstorder_Mean"; F6:"lbp.3D.m2_glszm_ZoneVariance"; F7: "wavelet.LHL_gldm_DependenceEntropy"; Radscore, radiomics score; GGO, ground-glass opacities; IIT, intralobular interstitial thickening. 0: negative, 1: positive in GGO and IIT, or 0: central, 1: peripheral, 2: mix in distribution

The model predicting performance of the radiomics model, CT sign model and combined model are summarized in Table 1. The ROC curves for each model are shown in Fig. 6a. After combining the radiomics feature and CT signs, the AUC values of were higher [0.959(95% CI 0.930–0.987)] than that of radiomics model [0.888(95% CI 0.8335–0.9417)] or CT sign model alone [0.906(95% CI 0.86–0.953)]. The AUC was compared between each paired model by Delong test (as shown in Additional file 8: Table 5). The AUC of combined model was significantly improved compared with radiomics model (*P* = 0.002) or CT sign (*P* = 0.004) alone. While there was no significant difference between radiomics model and CT sign model (*P* = 0.5916). Moreover, the accuracy, sensitivity, specificity, precision, positive prediction, and negative prediction of radiomics features with CT signs are higher than that of radiomics features or CT features alone (Table 1). The calibration curves in Fig. 6b also shows a better agreement between the prediction and observation in combined model. The wide range of high-risk threshold (0–0.8) of the DCA curves in the combined model also indicated its clinical usefulness with standardized net benefits larger than 0.6, which is optimal compared to other two models. (Fig. 6c). Meanwhile, considering the applicability of the models, the nomogram for the radiomics model and combined model were also illustrated in Fig. 7. By using such nomograms, the Radscore or the risk score of combined models involving Radscore component could be easily estimated in each input patient.

The overall performance of radiomics model and CT sign model among 1000-times bootstrap are summarized in Table 2. The appearing frequency of each radiomics feature or CT sign retained in the logistic regression model during 1000-times bootstrap was illustrated in Additional file 1: Fig. 1. The respective optimism-corrected AUC (Radiomics: 0.827, CT sign: 0.886) and average optimism (Radiomics: 0.061, CT sign: 0.02) represents a relatively good reliability of the model established from the selected feature. In addition, as shown in Additional file 1: Fig. 1, the radiomics feature and CT signs in the model appeared over 600 times during 1000-times bootstrap, which also reflected the reliability of the feature selection. The classifiers which might be appropriate for small sample size (Naïve Bayes, K Nearest Neighbor, Decision Tree) were tested. The model performance for supplementary models was summarized in Additional file 9: Table 6. Among these modeling methods, KNN showed a better predicting performance. But as considering AUC and accuracy of KNN model was comparable with logistic regression model, the logistic regression model which could construct nomogram might have more clinical application potential.

## Discussion

Considering the similarity of period of year, clinical symptoms of COVID-19 and influenza pneumonia and the importance of differential diagnosis, previous studies have compared the clinical manifestations, routine blood tests and CT findings between COVID-19 and influenza pneumonia. Shen and Liu et al. found that the clinical manifestations of COVID-19 and influenza pneumonia were very similar, but the monocyte percentage increased and the eosinophil count decreased in COVID-19 patients, and the GGO of COVID-19 on the CT image distributed in the periphery of the lung [8, 34]. In addition, the progression of chest CT findings is closely related to the prognosis of COVID-19. With the decrease of pure GGO, the increase of consolidation, the expansion of the lesion area, and the appearance of crazy-paving pattern, the COVID-19 patient's prognosis becomes worse [35, 36]. Diagnosing COVID-19 in the early stage is also beneficial to improve the prognosis. This study systematically analyzed the differences in CT signs and radiomics features COVID-19 and influenza pneumonia within 7 days. Our research found that four signs and seven radiomics features are related to COVID-19 infection. The selected CT signs and radiomics features can be used to construct CT signs model, radiomics models and combined model to distinguish COVID-19 and influenza pneumonia. In this study, the diagnostic performance of the radiomics model was not significantly better than the radiologists’ subjective judgments. However, the combined model which was based on CT signs and radiomics features, can distinguish COVID-19 from influenza pneumonia better than CT signs or radiomics features alone. And the combined model showed excellent and encouraging performance. The CT signs of COVID-19 and influenza pneumonia were compared in this study. We found that peripheral lesion, GGO, intralobular interstitial thickening and halo sign of COVID-19 pneumonia are more common than influenza pneumonia, which is consistent with previous studies [12, 13, 22, 37]. And the performance of CT signs to identify COVID-19 and influenza pneumonia is acceptable, which is consistent with Bai et al. (Accuracy 60–83%) [38]. The results of the radiologist's subjective evaluation showed that CT signs are of clinical value in identifying viral pneumonia. Radiomics is the generation of minable high throughput data through conversion of digital medical images (e.g. CT, MRI, PET/CT, and Ultrasound) [15]. In previous studies, radiomics had outstanding performance in the diagnosis, staging, prognosis, and treatment response prediction of tumors [15–17]. In addition, radiomics can give rise to a deeper understanding of the heterogeneity of pneumonia lesions [21, 39, 40]. Therefore, radiomics is theoretically a feasible method to distinguish COVID-19 pneumonia from influenza pneumonia. In our study, we selected seven of the most predictive radiological features, and most of them were filtered or transformed first-order or texture features. It might indicate that the distinguishment between such highly imaging overlapped pneumonia may need the emphasized features in the spatial or frequency domains or the relatively higher stability of these higher-order features. In clinical cancer research, radiomics features have been shown to reflect tumor invasiveness, malignancy, and lymph node metastasis potential and other biological characteristics [41–43]. However, we speculate that the cause of CT image heterogeneity between COVID-19 and influenza pneumonia may be different from the tumor. Subsequently, the radiomics prediction model was constructed. The performance of the classifier was 86.5% sensitivity, 78.4% specificity, 83.1% accuracy. In addition, the ROC curve was used for performance evaluation. The AUC was 0.888, indicating a relatively good performance.

In previous study, multiple CNN models were used to distinguish COVID-19 and influenza A pneumonia, And the Noisy-or Bayesian function model can make the accuracy reach 85.0% [14]. Zeng et al. used radiomics model to distinguish COVID-19 and influenza A pneumonia [44], and obtained an AUC (0.87) similar to ours. In this study, we selected significant features from more radiomics features (1316 in total), and included influenza A pneumonia and influenza B pneumonia, for which differential diagnosis is more difficult. Besides, in order to further improve the performance of the prediction model, we combined the radiologist's subjective visual assessment and computed radiomics features to construct the prediction model. It was found that the combined model has higher sensitivity, specificity, accuracy and AUC (0.959) than CT signs or radiomics model. The calibration curve and decision curve also showed that the reliability and stability of the combined prediction model were better. Shiri etal. used different radiomic features, feature selection and classifiers of multimodal images to construct prediction models and observed their performance in predicting the mutation status of EGFR and KRAS in non-small cell lung cancer [45]. The results show that the radiomic features extracted from different image feature sets can not only be used to predict the mutation status of EGFR and KRAS, but also have higher predictive power than conventional images. In addition, other previous studies have also shown that the combination of feature selection method and classification method can improve the predictive or prognostic performance of the model [46–49]. With the expansion of the study population and feature scale, the combination of meaningful biological information, clinical data and imaging omics may further improve prediction or prognostic performance. In addition, it is very important to select features and establish models according to different diseases, and to perform correlation analysis between radiomics features and more physiological and pathological features. Exploring the meaning of radiomics features in physiological or pathological mechanisms can make better use of radiomics features [45, 50].

This study has some limitations. First, as a retrospective study, there may be selection bias. But the results of our preliminary study are encouraging and will be verified in future larger studies. In addition, because of the small size of other single cases of pneumonia, we did not compare the characteristics of different viral pneumonia. Finally, the response of the lung to the virus is highly related to the host factor. CT data alone cannot completely distinguish the type of viral pneumonia, and more clinical features and laboratory examination data need to be considered. Combined with more clinical data, the predictive model may be better at identifying viral pneumonia.

In conclusion, we determined the chest CT signs and radiomics features that distinguished COVID-19 from influenza pneumonia and developed an effective predictive model. Our research shows that CT signs and radiomics features are effective tools for identifying COVID-19 and influenza pneumonia.

## Supplementary Information

**Additional file 1** Figure 1. The appearing frequency of (A) radiomics features and (B) CT signs among 1000-times bootstrap

**Additional file 2** Material 1. The equations of Radscore, CT sign and combined model.

**Additional file 3** Material 2. The method description for bootstrap validation for radiomics and CT sign models.

**Additional file 4** Table 1. Hospital name and number of cases in this multicenter study.

**Additional file 5** Table 2. Comparison of CT findings of COVID-19 and influenza pneumonia.

**Additional file 6** Table 3. The univariate and multivariate logistic regression for CT signs.

**Additional file 7** Table 4. The statistical difference of each selected radiomics features between COVID-19 and influenza groups.

**Additional file 8** Table 5. The Delong test between the AUC of each paired models.

**Additional file 9** Table 6. The performance of different classifiers for classifying COVID-19 and influenza.

## Data Availability

The data supporting this article are available from the corresponding author on reasonable request.

## Abbreviations

CT: computed tomography
COVID-19: coronavirus disease 2019
RT-PCR: real-time reverse transcription-polymerase chain reaction
GGO: ground-glass opacities
VOI: volume of interest
GLCM: gray level co‐occurrence matrix
GLSZM: gray level size zone matrix
GLRLM: gray level run length matrix
GLDM: gray level dependence matrix
NGTDM: neighboring gray tone difference matrix
LBP: local binary pattern
LASSO: least absolute shrinkage and selection operator
DCA: decision curve analysis
ROC: receiver operating characteristic
AUC: area under the curve

## References

[1] Zhu N, Zhang D, Wang W, Li X, Yang B, Song J, Zhao X, Huang B, Shi W, Lu R (2020). A novel coronavirus from patients with Pneumonia in China, 2019. N Engl J Med.

[2] Guan WJ, Ni ZY, Hu Y, Liang WH, Ou CQ, He JX, Liu L, Shan H, Lei CL, Hui DSC (2020). Clinical characteristics of coronavirus disease 2019 in China. N Engl J Med.

[3] Qiu H, Wu J, Hong L, Luo Y, Song Q, Chen D (2020). Clinical and epidemiological features of 36 children with coronavirus disease 2019 (COVID-19) in Zhejiang, China: an observational cohort study. Lancet Infect Dis.

[4] Chen N, Zhou M, Dong X, Qu J, Gong F, Han Y, Qiu Y, Wang J, Liu Y, Wei Y (2020). Epidemiological and clinical characteristics of 99 cases of 2019 novel coronavirus pneumonia in Wuhan, China: a descriptive study. Lancet.

[5] Fowlkes A, Steffens A, Temte J, Lonardo SD, McHugh L, Martin K, Rubino H, Feist M, Davis C, Selzer C (2015). Incidence of medically attended influenza during pandemic and post-pandemic seasons through the Influenza Incidence Surveillance Project, 2009–13. Lancet Respir Med.

[6] Moriyama M, Hugentobler WJ, Iwasaki A (2020). Seasonality of respiratory viral infections. Annu Rev Virol.

[7] Li Y, Reeves RM, Wang X, Bassat Q, Brooks WA, Cohen C, Moore DP, Nunes M, Rath B, Campbell H (2019). Global patterns in monthly activity of influenza virus, respiratory syncytial virus, parainfluenza virus, and metapneumovirus: a systematic analysis. Lancet Glob Health.

[8] Liu F, Zhang Q, Huang C, Shi C, Wang L, Shi N, Fang C, Shan F, Mei X, Shi J (2020). CT quantification of pneumonia lesions in early days predicts progression to severe illness in a cohort of COVID-19 patients. Theranostics.

[9] Fang Y, Zhang H, Xie J, Lin M, Ying L, Pang P, Ji W (2020). Sensitivity of chest CT for COVID-19: comparison to RT-PCR. Radiology.

[10] Chung M, Bernheim A, Mei X, Zhang N, Huang M, Zeng X, Cui J, Xu W, Yang Y, Fayad ZA (2020). CT imaging features of 2019 novel coronavirus (2019-nCoV). Radiology.

[11] Caruso D, Zerunian M, Polici M, Pucciarelli F, Polidori T, Rucci C, Guido G, Bracci B, De Dominicis C, Laghi A (2020). Chest CT features of COVID-19 in ROME Italy. Radiology.

[12] Liu M, Zeng W, Wen Y, Zheng Y, Lv F, Xiao K (2020). COVID-19 pneumonia: CT findings of 122 patients and differentiation from influenza pneumonia. Eur Radiol.

[13] Wang H, Wei R, Rao G, Zhu J, Song B (2020). Characteristic CT findings distinguishing 2019 novel coronavirus disease (COVID-19) from influenza pneumonia. Eur Radiol.

[14] Xu X, Jiang X, Ma C, Du P, Li X, Lv S, Yu L, Ni Q, Chen Y, Su J (2020). A deep learning system to screen novel coronavirus disease 2019 pneumonia. Eng (Beijing).

[15] Lambin P, Leijenaar RTH, Deist TM, Peerlings J, de Jong EEC, van Timmeren J, Sanduleanu S, Larue R, Even AJG, Jochems A (2017). Radiomics: the bridge between medical imaging and personalized medicine. Nat Rev Clin Oncol.

[16] Aerts HJ, Velazquez ER, Leijenaar RT, Parmar C, Grossmann P, Carvalho S, Bussink J, Monshouwer R, Haibe-Kains B, Rietveld D (2014). Decoding tumour phenotype by noninvasive imaging using a quantitative radiomics approach. Nat Commun.

[17] Yip SS, Aerts HJ (2016). Applications and limitations of radiomics. Phys Med Biol.

[18] Wynants L, Van Calster B, Collins GS, Riley RD, Heinze G, Schuit E, Bonten MMJ, Damen JAA, Debray TPA, De Vos M (2020). Prediction models for diagnosis and prognosis of covid-19 infection: systematic review and critical appraisal. BMJ.

[19] Chen Y, Wang Y, Zhang Y, Zhang N, Zhao S, Zeng H, Deng W, Huang Z, Liu S, Song B (2020). A quantitative and radiomics approach to monitoring ARDS in COVID-19 patients based on chest CT: a retrospective cohort study. Int J Med Sci.

[20] Wu Q, Wang S, Li L, Wu Q, Qian W, Hu Y, Li L, Zhou X, Ma H, Li H (2020). Radiomics analysis of computed tomography helps predict poor prognostic outcome in COVID-19. Theranostics.

[21] Zhang T, Yuan M, Zhong Y, Zhang YD, Li H, Wu JF, Yu TF (2019). Differentiation of focal organising pneumonia and peripheral adenocarcinoma in solid lung lesions using thin-section CT-based radiomics. Clin Radiol.

[22] Shi H, Han X, Jiang N, Cao Y, Alwalid O, Gu J, Fan Y, Zheng C (2020). Radiological findings from 81 patients with COVID-19 pneumonia in Wuhan, China: a descriptive study. Lancet Infect Dis.

[23] Mackin D, Fave X, Zhang L, Yang J, Jones AK, Ng CS, Court L (2017). Harmonizing the pixel size in retrospective computed tomography radiomics studies. PLoS ONE.

[24] Wei W, Hu XW, Cheng Q, Zhao YM, Ge YQ (2020). Identification of common and severe COVID-19: the value of CT texture analysis and correlation with clinical characteristics. Eur Radiol.

[25] van Griethuysen JJM, Fedorov A, Parmar C, Hosny A, Aucoin N, Narayan V, Beets-Tan RGH, Fillion-Robin JC, Pieper S, Aerts H (2017). Computational radiomics system to decode the radiographic phenotype. Cancer Res.

[26] Larue R, van Timmeren JE, de Jong EEC, Feliciani G, Leijenaar RTH, Schreurs WMJ, Sosef MN, Raat F, van der Zande FHR, Das M (2017). Influence of gray level discretization on radiomic feature stability for different CT scanners, tube currents and slice thicknesses: a comprehensive phantom study. Acta Oncol.

[27] Gu D, Hu Y, Ding H, Wei J, Chen K, Liu H, Zeng M, Tian J (2019). CT radiomics may predict the grade of pancreatic neuroendocrine tumors: a multicenter study. Eur Radiol.

[28] Wang X, Zhao X, Li Q, Xia W, Peng Z, Zhang R, Li Q, Jian J, Wang W, Tang Y (2019). Can peritumoral radiomics increase the efficiency of the prediction for lymph node metastasis in clinical stage T1 lung adenocarcinoma on CT?. Eur Radiol.

[29] Moons KG, Altman DG, Reitsma JB, Ioannidis JP, Macaskill P, Steyerberg EW, Vickers AJ, Ransohoff DF, Collins GS (2015). Transparent reporting of a multivariable prediction model for individual prognosis or diagnosis (TRIPOD): explanation and elaboration. Ann Intern Med.

[30] Battersby NJ, Bouliotis G, Emmertsen KJ, Juul T, Glynne-Jones R, Branagan G, Christensen P, Laurberg S, Moran BJ (2018). Development and external validation of a nomogram and online tool to predict bowel dysfunction following restorative rectal cancer resection: the POLARS score. Gut.

[31] Huang YQ, Liang CH, He L, Tian J, Liang CS, Chen X, Ma ZL, Liu ZY (2016). Development and validation of a radiomics nomogram for preoperative prediction of lymph node metastasis in colorectal cancer. J Clin Oncol.

[32] Ma X, Wang H, Huang J, Geng Y, Jiang S, Zhou Q, Chen X, Hu H, Li W, Zhou C (2020). A nomogramic model based on clinical and laboratory parameters at admission for predicting the survival of COVID-19 patients. BMC Infect Dis.

[33] Yu Y, Wang X, Li M, Gu L, Xie Z, Gu W, Xu F, Bao Y, Liu R, Hu S (2020). Nomogram to identify severe coronavirus disease 2019 (COVID-19) based on initial clinical and CT characteristics: a multi-center study. BMC Med Imaging.

[34] Shen C, Tan M, Song X, Zhang G, Liang J, Yu H, Wang C (2020). Comparative analysis of early-stage clinical features between COVID-19 and influenza A H1N1 virus pneumonia. Front Public Health.

[35] Pan F, Ye T, Sun P, Gui S, Liang B, Li L, Zheng D, Wang J, Hesketh RL, Yang L (2020). Time course of lung changes at chest CT during recovery from coronavirus disease 2019 (COVID-19). Radiology.

[36] Li K, Wu J, Wu F, Guo D, Chen L, Fang Z, Li C (2020). The clinical and chest CT features associated with severe and critical COVID-19 pneumonia. Invest Radiol.

[37] Tang X, Du RH, Wang R, Cao TZ, Guan LL, Yang CQ, Zhu Q, Hu M, Li XY, Li Y (2020). Comparison of hospitalized patients with ARDS caused by COVID-19 and H1N1. Chest.

[38] Bai HX, Hsieh B, Xiong Z, Halsey K, Choi JW, Tran TML, Pan I, Shi LB, Wang DC, Mei J (2020). Performance of radiologists in differentiating COVID-19 from non-COVID-19 viral pneumonia at chest CT. Radiology.

[39] Wang B, Li M, Ma H, Han F, Wang Y, Zhao S, Liu Z, Yu T, Tian J, Dong D (2019). Computed tomography-based predictive nomogram for differentiating primary progressive pulmonary tuberculosis from community-acquired pneumonia in children. BMC Med Imaging.

[40] Yanling W, Duo G, Zuojun G, Zhongqiang S, Yankai W, Shan L, Hongying C (2019). Radiomics nomogram analyses for differentiating pneumonia and acute paraquat lung injury. Sci Rep.

[41] Cho HH, Lee G, Lee HY, Park H (2020). Marginal radiomics features as imaging biomarkers for pathological invasion in lung adenocarcinoma. Eur Radiol.

[42] Takahashi S, Takahashi W, Tanaka S, Haga A, Nakamoto T, Suzuki Y, Mukasa A, Takayanagi S, Kitagawa Y, Hana T (2019). Radiomics analysis for glioma malignancy evaluation using diffusion kurtosis and tensor imaging. Int J Radiat Oncol Biol Phys.

[43] Wu S, Zheng J, Li Y, Wu Z, Shi S, Huang M, Yu H, Dong W, Huang J, Lin T (2018). Development and validation of an MRI-based radiomics signature for the preoperative prediction of lymph node metastasis in bladder cancer. EBioMedicine.

[44] Zeng QQ, Zheng KI, Chen J, Jiang ZH, Tian T, Wang XB, Ma HL, Pan KH, Yang YJ, Chen YP, et al. Radiomics-based model for accurately distinguishing between severe acute respiratory syndrome associated coronavirus 2 (SARS-CoV-2) and influenza A infected pneumonia. MedComm (Beijing) 2020. 10.1002/mco2.14 PMC7436469 32838396

[45] Shiri I, Maleki H, Hajianfar G, Abdollahi H, Ashrafinia S, Hatt M, Zaidi H, Oveisi M, Rahmim A (2020). Next-generation radiogenomics sequencing for prediction of EGFR and KRAS mutation status in NSCLC patients using multimodal imaging and machine learning algorithms. Mol Imaging Biol.

[46] Zhang B, He X, Ouyang F, Gu D, Dong Y, Zhang L, Mo X, Huang W, Tian J, Zhang S (2017). Radiomic machine-learning classifiers for prognostic biomarkers of advanced nasopharyngeal carcinoma. Cancer Lett.

[47] Hajianfar G, Shiri I, Maleki H, Oveisi N, Haghparast A, Abdollahi H, Oveisi M (2019). Noninvasive O(6) methylguanine-DNA methyltransferase status prediction in glioblastoma multiforme cancer using magnetic resonance imaging radiomics features: univariate and multivariate radiogenomics analysis. World Neurosurg.

[48] Leger S, Zwanenburg A, Pilz K, Lohaus F, Linge A, Zöphel K, Kotzerke J, Schreiber A, Tinhofer I, Budach V (2017). A comparative study of machine learning methods for time-to-event survival data for radiomics risk modelling. Sci Rep.

[49] Rastegar S, Vaziri M, Qasempour Y, Akhash MR, Abdalvand N, Shiri I, Abdollahi H, Zaidi H (2020). Radiomics for classification of bone mineral loss: a machine learning study. Diagn Interv Imaging.

[50] Sun R, Limkin EJ, Vakalopoulou M, Dercle L, Champiat S, Han SR, Verlingue L, Brandao D, Lancia A, Ammari S (2018). A radiomics approach to assess tumour-infiltrating CD8 cells and response to anti-PD-1 or anti-PD-L1 immunotherapy: an imaging biomarker, retrospective multicohort study. Lancet Oncol.

