# Supplementary figures and images for: CT-based radiomics combined with signs: a valuable tool to help radiologist discriminate COVID-19 and influenza pneumonia
Source: BMC Med Imaging. 2021 Feb 17;21:31. doi: 10.1186/s12880-021-00564-w (PMC7887546; doi:10.1186/s12880-021-00564-w)

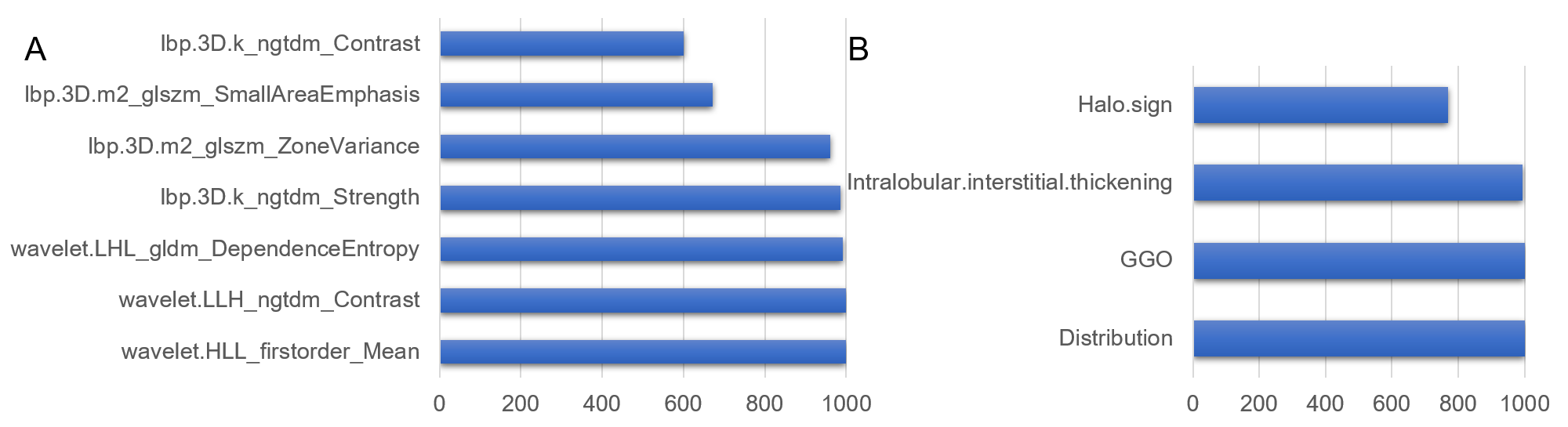

Supplement: Supplementary file 1 — Additional file 1 Figure 1. The appearing frequency of (A) radiomics features and (B) CT signs among 1000-times bootstrap [file 12880_2021_564_MOESM1_ESM.tif]
