# Supplementary material for: CT-based radiomics combined with signs: a valuable tool to help radiologist discriminate COVID-19 and influenza pneumonia
Source: BMC Med Imaging. 2021 Feb 17;21:31. doi: 10.1186/s12880-021-00564-w (PMC7887546; doi:10.1186/s12880-021-00564-w)
Supplement: Supplementary file 2 — Additional file 2 Material 1. The equations of Radscore, CT sign and combined model. [file 12880_2021_564_MOESM2_ESM.docx]

**Supplementary Material 1. The risk score formula of radiomics, CT sign and combined model.
The radiomics score (Radscore) was constructed based on the following regression formula:**

Radscore = 0.836 - 0.378*lbp.3D.k_ngtdm_Contrast + 2.973*lbp.3D.k_ngtdm_Strength - 0.423* lbp.3D.m2_glszm_SmallAreaEmphasis - 2.718*wavelet.LLH_ngtdm_Contrast –2.669* wavelet.HLL_firstorder_Mean -1.085* lbp.3D.m2_glszm_ZoneVariance -1.201*wavelet.LHL_gldm_DependenceEntropy

**The risk score formula of the CT sign model and the combined model are listed as follows.**

Score^CTsign^ = -4.719+2.656*Distribution + 2.864*GGO + 1.918*Intralobular.interstitial.thickening + 1.082*Halo.sign

Score^CTsign+Rad^ = -4.374+2.218*Distribution + 2.080*GGO + 2.538*Intralobular.interstitial.thickening + 1.036*Halo.sign+0.945*Radscore
