# Supplementary material for: CT-based radiomics combined with signs: a valuable tool to help radiologist discriminate COVID-19 and influenza pneumonia
Source: BMC Med Imaging. 2021 Feb 17;21:31. doi: 10.1186/s12880-021-00564-w (PMC7887546; doi:10.1186/s12880-021-00564-w)
Supplement: Supplementary file 3 — Additional file 3 Material 2. The method description for bootstrap validation for radiomics and CT sign models. [file 12880_2021_564_MOESM3_ESM.docx]

**Supplementary Material 2. The method description for bootstrap validation for radiomics and CT sign models.**

Step 1: Apparent performance. Taking the AUC of predicting model developed in original whole dataset as the apparent AUC.

Step 2: BOOTSTRAP sample splitting. The whole dataset was repeatedly split into training and test sets. The training set with the same sample size as original dataset was constructed by sampling with replacement from the original sample. The out-of-bag samples constructed the test set in each bootstrap.

Step 3: BOOTSTRAP model establishment. Starting features were features used to establish radiomics model and CT sign model based on the original whole dataset. And these features were further selected by backward stepwise logistic regression method with minimum AIC (the same as the modeling method in the manuscript) in each bootstrapped training set.

Step 4: BOOTSTRAP model establishment. The logistic regression model established from the training set was respectively tested in the “out-of-bag” test set and original dataset. The AUC were obtained for training and test set in each bootstrap loop.

Step 5: Model optimism among bootstrap. Calculate the model optimism as the difference between the bootstrap training set AUC and the test AUC.

Step 6: The optimism-corrected performance. Repeating Step 2 to Step 5 for 1000 times. The appearing frequency of each feature and the average optimism were calculated and recorded. Subtract the value from the apparent performance in step 1 and obtain an optimism-corrected performance.
