# Supplementary material for: CT-based radiomics combined with signs: a valuable tool to help radiologist discriminate COVID-19 and influenza pneumonia
Source: BMC Med Imaging. 2021 Feb 17;21:31. doi: 10.1186/s12880-021-00564-w (PMC7887546; doi:10.1186/s12880-021-00564-w)
Supplement: Supplementary file 4 — Additional file 4 Table 1. Hospital name and number of cases in this multicenter study. [file 12880_2021_564_MOESM4_ESM.docx]

**Supplementary Table 1. Hospital name and number of cases in this multicenter study**

| Hospital name | Total | Influenza pneumonia | COVID-19 |
| --- | --- | --- | --- |
| Kunming Third People’s Hospital | 45 | 21 | 24 |
| Yunnan Provincial Infectious Disease Hospital | 26 | 14 | 12 |
| Kunming First People’s Hospital | 6 | 6 | 0 |
| The First People’s Hospital of Zhaotong | 19 | 4 | 15 |
| Third People’s Hospital of Yunnan Province | 11 | 11 | 0 |
| Xishuangbanna People's Hospital | 11 | 4 | 7 |
| Qujing First People’s Hospital | 8 | 0 | 8 |
| Dali People's Hospital | 7 | 0 | 7 |
| Yuxi People's Hospital | 5 | 0 | 5 |
| Lijiang People's Hospital | 3 | 0 | 3 |
| Second People’s Hospital of Yunnan Province | 3 | 3 | 0 |
| Chuxiong People's Hospital | 3 | 0 | 3 |
| Lancang First People’s Hospital | 3 | 0 | 3 |
| First People’s Hospital of Yunnan Province | 2 | 2 | 0 |
| Baoshan People's Hospital | 2 | 0 | 2 |
| Total | 154 | 65 | 89 |

Note: COVID-19, coronavirus disease 2019. Data are number of patients.
