# Supplementary material for: CT-based radiomics combined with signs: a valuable tool to help radiologist discriminate COVID-19 and influenza pneumonia
Source: BMC Med Imaging. 2021 Feb 17;21:31. doi: 10.1186/s12880-021-00564-w (PMC7887546; doi:10.1186/s12880-021-00564-w)
Supplement: Supplementary file 5 — Additional file 5 Table 2. Comparison of CT findings of COVID-19 and influenza pneumonia. [file 12880_2021_564_MOESM5_ESM.docx]

**Supplementary Table 2. Comparison of CT findings of COVID-19 and influenza pneumonia**

| CT findings | Influenza pneumonia (n=65) | COVID-19 (n=89) | P value |
| --- | --- | --- | --- |
| Lesion distribution |  |  | <0.001* |
| Central | 0(0.00%) | 1(1.12%) |  |
| Peripheral | 30(46.15%) | 76(85.39%) |  |
| Mix | 35(53.85%) | 12(13.48%) |  |
| Lobe predomination |  |  |  |
| Upper left lobe | 43(66.15%) | 52(58.43%) | 0.33 |
| Left lower lobe | 47(72.31%) | 60(67.42%) | 0.515 |
| Right upper lobe | 44(67.69%) | 48(53.93%) | 0.086 |
| Right middle lobe | 34(52.31%) | 35(39.33%) | 0.11 |
| Right lower lobe | 54(83.08%) | 68(76.40%) | 0.313 |
| Main features |  |  |  |
| GGO | 30(46.15%) | 83(93.26%) | <0.001* |
| Partial consolidation | 31(47.69%) | 33(37.08%) | 0.187 |
| Consolidation | 11(16.92%) | 3(3.37%) | 0.004* |
| Bronchiectasis | 21(32.31%) | 20(22.47%) | 0.173 |
| Bronchial wall thickening | 14(21.54%) | 9(10.11%) | 0.049* |
| Tree in bud | 18(27.69%) | 0(0.00%) | <0.001* |
| Crazy paving pattern | 6(9.23%) | 2(2.25%) | 0.118 |
| Air bronchogram | 9(13.85%) | 8(8.99%) | 0.342 |
| Septal thickening | 29(44.62%) | 32(35.96%) | 0.278 |
| Intralobular interstitial thickening | 15(23.08%) | 51(57.30%) | <0.001* |
| Halo sign | 14(21.54%) | 44(49.44%) | <0.001* |
| Reversed halo sign | 0(0.00%) | 2(2.25%) | 0.509 |
| Mediastinal lymphadenectasis | 7(10.77%) | 1(1.12%) | 0.022* |
| Pleural effusion | 20(30.77%) | 0(0.00%) | <0.001* |

Note: GGO, ground-glass opacities; COVID-19, coronavirus disease 2019. Categorical data are presented as n (%). *, compared with COVID-19, significance was considered when P < 0.05.
