# Supplementary material for: CT-based radiomics combined with signs: a valuable tool to help radiologist discriminate COVID-19 and influenza pneumonia
Source: BMC Med Imaging. 2021 Feb 17;21:31. doi: 10.1186/s12880-021-00564-w (PMC7887546; doi:10.1186/s12880-021-00564-w)
Supplement: Supplementary file 6 — Additional file 6 Table 3. The univariate and multivariate logistic regression for CT signs. [file 12880_2021_564_MOESM6_ESM.docx]

**Supplementary Table 3. The univariate and multivariate logistic regression for CT signs.**

|  | **Univariate** | | **Multivariate** | |
| --- | --- | --- | --- | --- |
| **CT sign** | **OR(95%CI)** | **Pvalue** | **OR(95CI)** | **Pvalue** |
| Distribution | 7.465(3.525-16.786) | <0.0001 | 14.237(5.243-44.458) | <0.0001 |
| GGO | 16.139(6.569-46.180) | <0.0001 | 17.525(5.844-63.522) | <0.0001 |
| Consolidation | 0.171(0.037-0.577) | 0.008 |  |  |
| Bronchial wall thickening | 0.41(0.159-1.003) | 0.054 |  |  |
| Tree in bud | 1.24E-08(NA-3.85E+16) | 0.984 |  |  |
| Intralobular interstitial thickening | 4.473(2.231-9.358) | <0.0001 | 6.806(2.522-20.908) | <0.0001 |
| Halo sign | 3.562(1.762-7.531) | 0.0005 | 2.951(1.103-8.478) | 0.035 |
| Mediastinal lymphadenectasis | 0.094(0.005-0.548) | 0.029 |  |  |
| Pleural effusion | 1.19E-08(NA-2.26E+14) | 0.983 |  |  |
