# Supplementary material for: CT-based radiomics combined with signs: a valuable tool to help radiologist discriminate COVID-19 and influenza pneumonia
Source: BMC Med Imaging. 2021 Feb 17;21:31. doi: 10.1186/s12880-021-00564-w (PMC7887546; doi:10.1186/s12880-021-00564-w)
Supplement: Supplementary file 7 — Additional file 7 Table 4. The statistical difference of each selected radiomics features between COVID-19 and influenza groups. [file 12880_2021_564_MOESM7_ESM.docx]

**Supplementary Table 4. The statistical difference of each selected radiomics features between COVID-19 and influenza groups.**

| **Radiomics feature name** | **Influenza** | **COVID-19** | **Statistics** | **P-value^b^** | **AUC (95%CI)^c^** | **Feature description** |
| --- | --- | --- | --- | --- | --- | --- |
| lbp.3D.k_ngtdm_Contrast | 0.29±1.12^a^ | -0.21±0.85 | 2.687 | 0.007 | 0.627(0.551-0.703) | **lbp.3D.k_ngtdm:** Neighbouring Gray Tone Difference Matrix (NGTDM) calculated from Local Binary Pattern (LBP)-filtered kurtosis image.  **lbp.3D.k_ngtdm_Contrast:** is a measure of the spatial intensity change of NGTDM texture matrix. The lower value in COVID-19 indicates a slow intensity change between pixels and their neighbor pixels in NGTDM texture matrix. |
| lbp.3D.k_ngtdm_Strength | -0.24±0.48 | 0.18±1.22 | -1.758 | 0.079 | 0.583(0.531-0.635) | **lbp.3D.k_ngtdm_Strength:** is a measure of the primitives in an NGTDM matrix from LBP-filtered kurtosis image. The higher value in COVID19 indicates more large coarse differences in gray level intensities. |
| lbp.3D.m2_glszm_SmallAreaEmphasis | 0.14±0.99 | -0.10±1.00 | 2.072 | 0.038 | 0.598(0.515-0.681) | **lbp.3D.m2_glszm_SmallAreaEmphasis:** is Gray Level Size Zone (GLSZM) texture. A lower value indicates relatively rough textures in COVID19. |
| wavelet.LLH_ngtdm_Contrast | 0.20±1.38 | -0.14±0.55 | 1.857 | 0.063 | 0.588(0.504-0.672) | **wavelet.LLH_ngtdm:** is NGTDM texture matrix calculated from 3D wavelet decomposed image using LLH filter channel.  **wavelet.LLH_ngtdm_Contrast:** is a measure of the spatial intensity change of NGTDM texture matrix. The lower value in COVID-19 indicates a slow intensity change between pixels and their neighbor pixels in NGTDM texture matrix. |
| wavelet.HLL_firstorder_Mean | 0.18±0.73 | -0.13±1.14 | 4.377 | <0.001 | 0.707(0.622-0.792) | **wavelet.HLL_firstorder_mean:** is the mean intensity calculated from 3D wavelet decomposed image using in HLL filter channel. The lower value in COVID19 represents a lower intensity. |
| lbp.3D.m2_glszm_ZoneVariance | 0.27±1.46 | -0.20±0.29 | 3.38 | 0.001 | 0.66(0.572-0.747) | **lbp.3D.m2_glszm_ZoneVariance:** is a measure of zone variance in Gray Level Size Zone (GLSZM) texture matrix from LBP-filterd image (level 2 in spherical harmonics). The lower value in COVID19 indicates a slower changing texture or relative flat texture in local zone. |
| wavelet.LHL_gldm_DependenceEntropy | 0.23±0.93 | -0.17±1.02 | 3.505 | <0.001 | 0.666(0.577-0.754) | **wavelet.LHL_gldm_DependenceEntropy:** is a measure of DependenceEntropy in Gray Level Dependence Matrix (GLDM) texture from 3D-wavelet decomposed images using LHL filter channel. The lower value in COVID19 indicated a less complicated texture distribution. |

1. Mean ± SD;
2. Statistical significance level: P<0.05
3. The AUC calculated from individual radiomics feature.
