# Supplementary material for: CT-based radiomics combined with signs: a valuable tool to help radiologist discriminate COVID-19 and influenza pneumonia
Source: BMC Med Imaging. 2021 Feb 17;21:31. doi: 10.1186/s12880-021-00564-w (PMC7887546; doi:10.1186/s12880-021-00564-w)
Supplement: Supplementary file 8 — Additional file 8 Table 5. The Delong test between the AUC of each paired models. [file 12880_2021_564_MOESM8_ESM.docx]

**Supplementary Table 5. The Delong test between the AUC of each paired models.**

| **Compared group** | Z | Pvalue |
| --- | --- | --- |
| **Radiomics model vs CTsign model** | -0.53653 | 0.5916 |
| **Radiomics model vs CTsign_+radiomcis model** | -3.127 | 0.002 |
| **CT sign model vs CTsign_+radiomcis model** | -2.8829 | 0.004 |
