# Supplementary material for: CT-based radiomics combined with signs: a valuable tool to help radiologist discriminate COVID-19 and influenza pneumonia
Source: BMC Med Imaging. 2021 Feb 17;21:31. doi: 10.1186/s12880-021-00564-w (PMC7887546; doi:10.1186/s12880-021-00564-w)
Supplement: Supplementary file 9 — Additional file 9 Table 6. The performance of different classifiers for classifying COVID-19 and influenza. [file 12880_2021_564_MOESM9_ESM.docx]

**Supplementary Table 6. The performance of different classifiers for classifying COVID-19 and influenza.**

| **Performance index** | **Logistic regression** | **Naïve Bayes** | **K Nearest Neighbor** | **Decision Tree** |
| --- | --- | --- | --- | --- |
| **Parameters** |  | Gaussian Naïve Bayes | N neighbors= 6 | Max depth = 3;  min_samples_split = 2 |
| **AUC** | 0.888(0.8335-0.9417) | 0.8 (0.738, 0.858) | 0.897 (0.856, 0.935) | 0.838 (0.788, 0.886) |
| **Sensitivity** | 0.865 | 0.876 | 0.854 | 0.933 |
| **Specificity** | 0.784 | 0.4 | 0.831 | 0.692 |
| **positive prediction** | 0.846 | 0.667 | 0.874 | 0.806 |
| **negative prediction** | 0.809 | 0.703 | 0.806 | 0.882 |
| **Accuracy** | 0.831 | 0.675 | 0.844 | 0.831 |

Note: AUC, ﻿area under curve.
